# Phylogenetic Tracing of Evolutionarily Conserved Zonula Occludens Toxin Reveals a “High Value” Vaccine Candidate Specific for Treating Multi-Strain *Pseudomonas aeruginosa* Infections

**DOI:** 10.3390/toxins16060271

**Published:** 2024-06-14

**Authors:** Payam Benyamini

**Affiliations:** Department of Health Sciences at Extension, University of California Los Angeles, 1145 Gayley Ave., Los Angeles, CA 90024, USA; payamb@ucla.edu

**Keywords:** antigen vaccine, immunotherapy, infectious diseases, in silico drug design, Gram-negative bacteria, *Pseudomonas aeruginosa*, phylogenetics, zonula occludens toxin, zot, bioinformatics

## Abstract

Extensively drug-resistant *Pseudomonas aeruginosa* infections are emerging as a significant threat associated with adverse patient outcomes. Due to this organism’s inherent properties of developing antibiotic resistance, we sought to investigate alternative strategies such as identifying “high value” antigens for immunotherapy-based purposes. Through extensive database mining, we discovered that numerous Gram-negative bacterial (GNB) genomes, many of which are known multidrug-resistant (MDR) pathogens, including *P. aeruginosa*, horizontally acquired the evolutionarily conserved gene encoding Zonula occludens toxin (Zot) with a substantial degree of homology. The toxin’s genomic footprint among so many different GNB stresses its evolutionary importance. By employing in silico techniques such as proteomic-based phylogenetic tracing, in conjunction with comparative structural modeling, we discovered a highly conserved intermembrane associated stretch of 70 amino acids shared among all the GNB strains analyzed. The characterization of our newly identified antigen reveals it to be a “high value” vaccine candidate specific for *P. aeruginosa*. This newly identified antigen harbors multiple non-overlapping B- and T-cell epitopes exhibiting very high binding affinities and can adopt identical tertiary structures among the least genetically homologous *P. aeruginosa* strains. Taken together, using proteomic-driven reverse vaccinology techniques, we identified multiple “high value” vaccine candidates capable of eliciting a polarized immune response against all the *P. aeruginosa* genetic variants tested.

## 1. Introduction

Epithelial tissues comprise tightly woven sheets of avascular cells distributed throughout the body, covering both the internal and external surfaces, lining the deep layers of organs, and performing a variety of physiological functions [1,2,3,4,5,6,7,8,9]. Tight junctions (TJs) are the subcellular structures essential for maintaining a continuously interconnected epithelial monolayer [10,11,12,13]. TJs comprise a multiplex arrangement of both cytosolic and integral membrane proteins that physically interconnect adjacent epithelial cells, establishing an impenetrable seal [12,13,14,15,16,17,18,19,20,21]. Although epithelial tissues serve as strong protective barriers that prevent infectious agents from disseminating, numerous Gram-negative bacterial (GNB) pathogens have horizontally acquired an evolutionary conserved virulence factor; Zonula occludens toxin (Zot), capable of commandeering host-cell TJ multiplexes and mediating the paracellular transport into the deeper layers of host tissues [22,23,24,25,26]. The toxins highly conserved horizontal passage among multitudes of GNB, with a very high degree of homology, strongly emphasizing its vital importance in the evolution of GNB pathogenesis; specifically for dissemination purposes.

Zonula occludens toxin is encoded by a wide variety of GNB pathogens and displays properties that enhance the microbial permeation between epithelial monolayers. Zot is an outer membrane protein originally identified in the toxigenic GNB pathogen, *Vibrio cholerae* [22,24,26]. The toxin, along with a variety of other virulence factors, is predominantly part of a pathogenicity island encoded by the filamentous phage, CTXΦ, which is responsible for the horizontal transfer of several virulence genes to *V. cholera* [25,27]. Zot displays host protein mimicry functions that regulate the microbial passage between physically linked epithelial cells. Based on the virulence properties exhibited by Zot, its main role in host–pathogen interaction is associated with inducing signaling cascades that control epithelial cell tight junctional complexes and mediate the paracellular passage into the deeper layers of tissue [22,23,26,28,29,30]. Affinity-purified anti-Zot antibodies show that the subcellular localization of *V. cholerae*-derived Zot is on the outer membrane and has a molecular mass of ~45 kDa. Additionally, a second immunoreactive protein analogous to the ~33 kDa amino terminus of Zot has also been identified to be membrane bound. The ~33 kDa transmembrane fragment is further processed at a cleavage site located at amino acid residue 287 [26]. The final biologically active toxigenic fragment (of ~12 kDa) localized to the carboxyl terminus is released into the host intestinal milieu, where it binds specific cell surface receptors present on the mature cells of small intestinal villi and initiates a signal transduction cascade to commandeer the dissociation of tight junctions [22]. In addition to being encoded by *V. cholerae* and many more GNB, genome annotation studies reveal the presence of Zot-encoding genes in the genomes of numerous *Pseudomonas aeruginosa* genetic variants. This ability to control paracellular transport by deploying Zot is what enables the highly invasive progression of *P. aeruginosa* into the deeper layers of tissue, and the subsequent dissemination to the distal anatomical regions.

*P. aeruginosa* produces a multitude of intrinsic and acquired virulence factors that enable it to adapt to its host. These virulence factors are deployed at different stages of disease, depending on the variations in the host’s environmental factors and immune defenses [31]. These virulence factors are grouped based on whether they are cytosolic effectors, membrane bound, or secreted into the extracellular milieu [32,33,34,35,36]. Cytosolic effector proteins are injected into host cells to rapidly commandeer host functions and actively regulate shifts in metabolism, physiochemistry, and morphology [37,38]. Membrane-bound factors mediate the physical host/pathogen interaction and biofilm formation [39,40,41,42,43]. Secreted toxins shape the overall pathogenic capacity of *P. aeruginosa*, by enabling it to carry out a multitude of functions that exploit its host. These secreted toxins exert their affects in a variety of ways such as molecular mimicry, directly damaging host cells, enzymatic hydrolysis, providing dissemination capabilities, diverting or interfering with the host’s inflammatory responses, and enhanced quorum sensing capabilities [31,36,44,45,46,47,48,49]. Traditionally, secreted virulence factors are commonly used as vaccine antigens for immunization strategies and display a minimal likelihood of the emergence of therapeutic resistance [36,50,51]. Accordingly, Zot’s highly pervasive characteristic and extensive homology shared amongst numerous GNB pathogens further stresses its importance in *P. aeruginosa* pathogenesis and serves as a “high value” vaccine candidate for the development of multi-strain immunotherapy-based treatment modalities.

It is estimated that bacterial infections account for approximately 7.7 million worldwide deaths annually [52,53]. Surgical sites represent the leading cause of infections, followed by urinary tract, bacteremia, pneumonia and other forms of bacterial diseases [52,53]. *P. aeruginosa* exhibits remarkable clinical importance as it accounts for 7% amongst all nosocomial infections and is the cause of 23% of all ICU-acquired infections [54]. *P. aeruginosa* is a ubiquitous, non-spore forming, aerobic, Gram-negative bacilli that primarily favors wet and humid niches [55]. It is an extensive MDR pathogen that causes a multitude of infections in both immunocompetent and immunocompromised hosts [56,57]. The pathogen can originate from either exogenous or endogenous sources, such as an individual’s microflora, central lines, catheters, ventilators, healthcare workers and other non-mechanical associated infection systems like enteric and bloodstream, which are the primary sources [57]. Second to the Gram-positive pathogen, *Staphylococcus aureus*, *P. aeruginosa* is the leading GNB pathogen identified among healthcare-associated pneumonia cases and accounts for ~26% of global infections [53]. The mortality due to ventilator-associated pneumonia secondary to *P. aeruginosa* is estimated to be as high as 32–42.8% [54]. *P. aeruginosa* is frequently associated with catheter infections, bacteremia, surgical site, burn wound and urinary tract infections (UTI) and is the predominant cause of morbidity and mortality in cystic fibrosis patients [54,58]. In its extreme form, uncontrolled *P. aeruginosa* infections are fatal [54]. These infections are generally managed with antibiotics; however, the rapid rise in MDR nosocomial strains has made them very difficult to treat [59]. Accordingly, we have sought to identify antibiotic independent strategies for preventing/treating MDR *P. aeruginosa*-associated disease.

Herein, we report a reverse vaccinology strategy utilizing proteomic-based phylogenetic tracing approaches to reconstruct the evolutionary history of Zot and assess its relatedness among a variety of different MDR-GNB pathogens [60,61,62,63,64]; these include, but are not limited to, *P. aeruginosa*, *Neisseria gonorrhoeae*, *Neisseria meningitidis*, *Acinetobacter baumannii*, *Klebsiella pneumoniae*, *Escherichia coli*, *Salmonella enterica*, *Yersinia pestis* and *V. cholerae*. First, we performed an extensive database mining analysis and identified the evolutionary conserved virulence factor, Zot, encoded by numerous MDR-GNB, including several *P. aeruginosa* genetic variants. Using *P. aeruginosa* as our model organism, we performed both nucleic acid and protein-based phylogenetics, in conjunction with utilizing sequence alignment and structural modeling to identify long stretches of evolutionary conserved amino acid sequences shared among all different *P. aeruginosa* strains queried. Accordingly, we identified a highly conserved loci, in the native Zot protein, comprised of an intermembrane-associated stretch of 70 amino acids with very high antigenic potential. Upon characterizing our newly discovered antigen, we noticed its properties to be classified as a “high value” vaccine candidate for the treatment of a multitude of MDR-GNB pathogens, including *P. aeruginosa* infections.

## 2. Results

**Figure 1:**
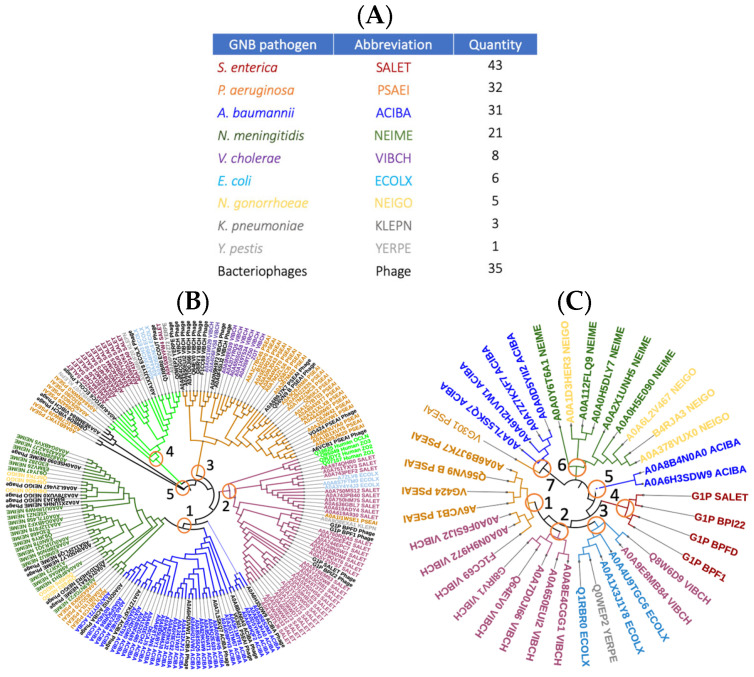
Numerous GNB genomes harbor evolutionarily conserved Zot homologs. Table representing 150 (UniProt confirmed) protein entities and their corresponding bacteriophages that harbor the Zot gene. Distribution among all pathogens falls under eight different GNB species (**A**). Proteomic-driven phylogenetics of eight GNB and their corresponding bacteriophages reveal that they are distributed among four clades (red circles # 1–4) and one outgroup lineage labeled # 5 (**B**). Phylogenetic relationship among species-specific bacteriophages falls into seven clads (**C**).

Proteomic mining identifies evolutionarily conserved Zonula occludens toxin homologs distributed throughout the numerous clinically relevant GNB pathogens. To trace the phylogenetic relationship of Zot among different GNB pathogens, the multiple protein sequence alignment algorithm, MUSCLE, was used [65]. First, we performed an extensive UniProt search with Zonula occludens toxin as the query (accessed 26 February 2024). Of the 284 predicted individual protein entities identified, the closest full-length matches (≥345 amino acids) to the *V. cholerae* Zot protein sequence were chosen. A total of 150 different protein entities and 35 species-specific bacteriophages were identified. All the identified protein entities were distributed among eight different clinically relevant MDR-GNB pathogens: *P. aeruginosa*, *V. cholerae*, *N. gonorrhoeae*, *N. meningitidis*, *A. baumannii*, *K. pneumoniae*, *E. coli*, *S. enterica* and *Y. pestis* (Figure 1A). It should be noted that the database mining results did not reveal the presence of Zot-like proteins associated with any Gram-positive bacteria, suggesting that the evolutionary path of Zot is only restricted to GNB pathogens. The different composition of Gram-positive vs. Gram-negative bacterial cell walls may be a contributing factor responsible for the restricted evolution of the toxin to the outer membrane of GNB pathogens.

Zot protein-mediated phylogenetic comparisons of the eight different pathogens revealed that they are all distributed into four different clades and two outgroup lineage (Figure 1B). Clade one is subdivided into two nested clades including *Neisseria* spp. and *A. baumannii*. Additionally, the *Neisseria* spp. nested clade also shares an ancestry with four different *P. aeruginosa* strains. Clade two shows a shared ancestry between the majority of different *S. enterica* strains and three of six identified *E. coli* strains. Moreover, one *K. pneumoniae* and one *P. aeruginosa* strains were also included in clade two. Interestingly, the phylogram shows that *S. enterica*-encoded Zot shares a high degree of homology with human-associated Zonula occludens (-1, -2, -3) and occludin proteins, which are also included in clade two. Clade three exhibits a shared ancestry between most *P. aeruginosa* strains and all identified *V. cholerae* stains. Clade four is the most diverse of all and includes a mixture of five different GNB pathogens. Specific strains of *P. aeruginosa*, *S. enterica*, *E. coli*, *K. pneumoniae* and *Y. pestis* were all found to be present in clade four. In contrast, the three other clades were more species-defined and not as diverse. In addition to the presence of the four different clades, there appears to be two outgroup lineages designated the number five on the phylogram (Figure 1B). These outgroup lineages only comprise a few *P. aeruginosa* strains and do not display any common ancestry with any of the four clades. To assess the contribution of bacteriophages to the horizontal transfer of Zot, several phages were identified and included in our phylogenetic studies. The results indicate the presence of multiple species-specific bacteriophages distributed throughout each clade and cluster (Figure 1A,B).

A comparative analysis of the phylogenetic relationship among different bacteriophages expressing Zot showed their evolutionary passage into 7 clads. For example, clad one comprises phages belonging to *V. cholerae* and all of *P. aeruginosa.* Clad two is restricted to only *V. cholerae* phages. Clad three contains *E. coli*, *V. cholerae* and *Y. pestis* phages. Clad four is only restricted to *S. enterica* phages. Clade five only has *A. baumannii* phages. Clad 6 has a mixture of *N. gonorrhoeae* and *N. meningitidis* phages. Lastly, clad seven is comprised mainly of *A. baumannii* phages and a single *P. aeruginosa* phage (Figure 1C).

**Figure 2:**
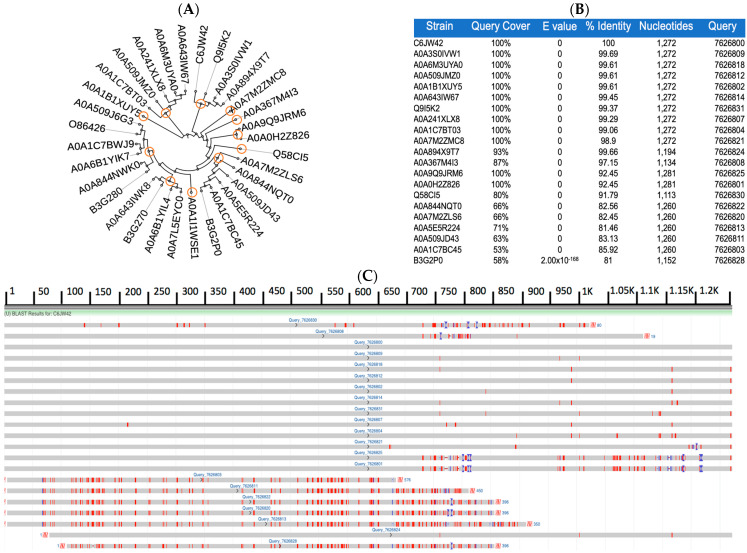

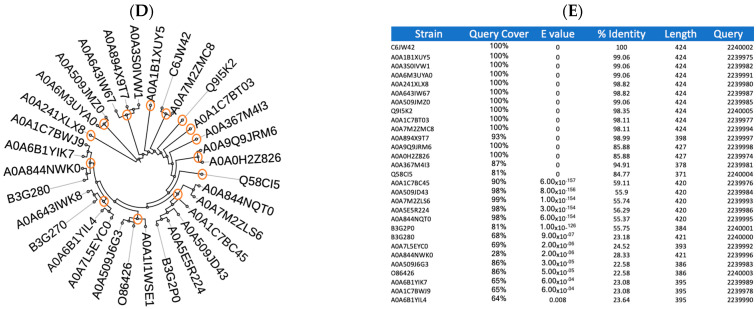
Phylogenetic tracing of *P. aeruginosa*-encoded Zot reveals a high degree of homology shared among both nucleotide and amino acid sequences. Nucleic acid-based phylogram of 32 different strains distributed among 21 unique clusters and 14 different clads (**A**). Nucleotide-based percentage homology and query coverage among different variants (**B**). Schematic representation of homo- (gray) and heterogeneous (red) sequences of the Zot gene, among all genetically diverse strains (**C**). Amino acid-based phylogram reveals 32 different strains distributed among 23 unique clusters and 11 different clads (**D**). Proteomic-based phylogenetic tracing of Zot among different strains shows varying degrees of homology, ranging from 20 to 100% identity (**E**). Percentage identity is calculated as the number of matches in an alignment row relative to the alignment length, where the alignment length is either the length as determined by MUSCLE-specific BLAST alignment or the aligned sequence length minus any gaps (for all other alignments). Percentage coverage is calculated as the number of aligned nucleotides or residues in an alignment row relative to the aligned length of the anchor or consensus sequence. Orange circles represent each clade.

The multiple sequence alignments of Zot reveal a high degree of nucleotide and amino acid sequence homology among many different *P. aeruginosa* genetic variants. Using our nucleic acid-based alignment data, we constructed a phylogenetic tree and traced the nucleotide distribution of the homo- and heterogeneous sequences of the Zot gene, among different *P. aeruginosa* strains. Our phylogenetic data show that 32 different strains are distributed among 21 unique clusters and 14 different clads. Of the 14 clades, 8 are nested and represent a total of 26 different strains. Furthermore, the phylogram revealed the presence of two out-group lineages comprising three different *P. aeruginosa* strains that do not show a close ancestry to any of the other 29 genetic variants (Figure 2A). Quantitative assessment of the nucleic acid sequence homology, between the 21 unique *P. aeruginosa* clusters, revealed a range between 81% and 100% sequence identity, with a query coverage of 58% to 100% (Figure 2B). A graphical representation of the nucleotide sequence homology among the different strains reveals very short, dispersed stretches of non-homologous regions (marked in red) located in the mid and N-terminal regions of the Zot gene, possibly displaying strain- and/or niche-specific nucleotide sequences (Figure 2C).

Our proteomic-based phylogenetic studies show that 32 different strains were distributed among 23 unique clusters and 11 different clads. Of the 11 clades, 6 are nested and represent a total of 26 different genetic variants. Additionally, the phylogram also revealed the presence of 2 outgroup lineages comprising 6 different *P. aeruginosa* strains that do not show any close ancestry to the other 27 variants (Figure 2D). The quantitative assessment of amino acid sequence homology showed a range between 22.58% and 100% identity, with query coverage of 28% to 100% (Figure 2E). Orange circles represent each clad.

**Figure 3:**
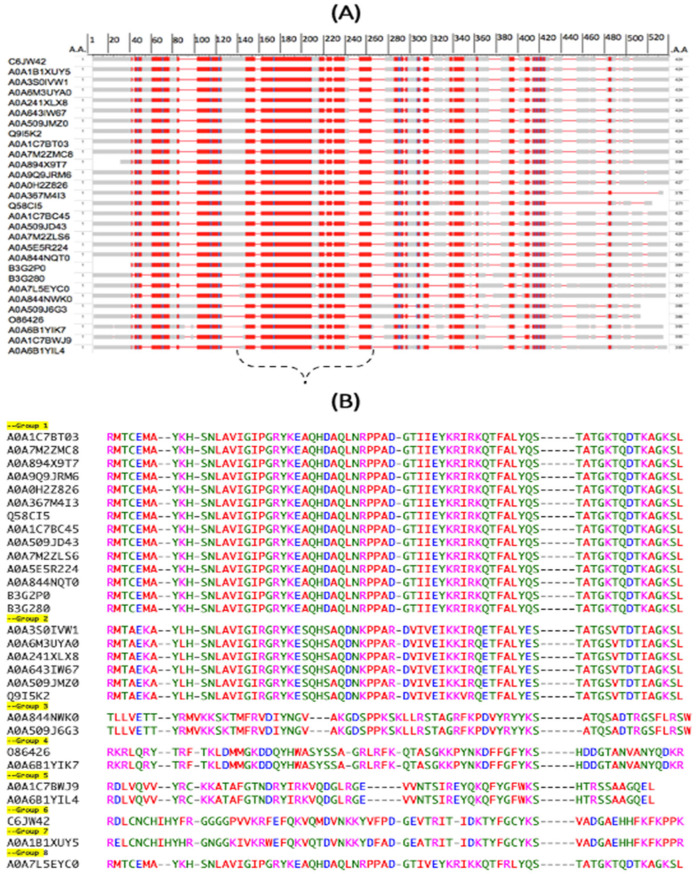
Identification of an evolutionary conserved stretch of 70 amino acids, among different *P. aeruginosa* strains, is located in the mid-left section of the Zot protein. The top view shows the length of amino acid coverage in the alignment. Positions where most sequences are homologous to the consensus sequence are colored in red, and positions that contain mismatches are colored in gray. Gaps are indicated with a faint red line, and insertions relative to the anchor sequence are indicated by blue (**A**). Amino acid sequence of evolutionarily conserved stretch of 70 Amino acids, encoded by different *P. aeruginosa* genetic variants (**B**). Amino acid color alignments are based according to similarities in amino acid properties. [green, polar uncharged; magenta, positively charged; red, hydrophobic; blue, negatively charged].

The proteomic-based sequence alignments of Zot identify the presence of a long stretch of 70, evolutionary conserved, amino acids shared among numerous *P. aeruginosa* genetic variants. Multiple sequence alignment tools are key components in predicting the protein structure and function properties and are ideal for identifying evolutionarily conserved stretches of amino acid sequences that can serve as “high value” vaccine candidates, capable of targeting the maximum number of *P. aeruginosa* strains. A total of 29 different *P. aeruginosa* genetic variants were analyzed in this study. Accordingly, our proteomic data of Zot reveals the presence of several stretches of highly conserved amino acid sequences dispersed though different regions of the toxin (red color). As can be seen in the graphical representation, there appears to be a very long stretch of highly conserved amino acid sequences positioned near the mid-left section of the Zot protein (Figure 3A). To further investigate this highly conserved region, we specifically chose the linear stretch of amino acids located between arginine 153 and leucine 223. The alignment of this newly identified 70 amino acid multi-strain antigen shows that all the strains are divided into eight subgroups, based on the sequence homology (Figure 3B). The number of strains per group is as follows: (1) 14 strains, (2) 6 strains, (3) 2 strains, (4) 2 strains, (5) 2 strains, (6) 2 strains, (7) 1 strain and (8) 1 strain.

It is well established that transmembrane proteins serve a significant role in maintaining proper cell functions such as mediating signal transduction, transmembrane transport, cell communication and most notably virulence [66]. Accordingly, transmembrane proteins are considered ideal vaccine candidates due to the exposure of highly immunogenic epitopes present on the outer membrane [67,68]. Most alleles encoding major histocompatibility complex I and II (MHC-I, -II) are generally hydrophobic in character, and the peptides derived from transmembrane proteins are predicted to be present more often. This high MHC binding capacity of transmembrane peptide domains is based on their displayed hydrophobic nature [67,68]. Furthermore, transmembrane proteins tend to be evolutionarily conserved among many different strains and across many different Gram-negative bacterial species, as is the case with our newly identified “high value” antigen. While most research is focused on identifying epitopes with specific antigenicity, we sought to determine in which domain our newly identified antigen is located. Such assessment will shed light on the immunogenic potential of our new vaccine candidate.

**Figure 4:**
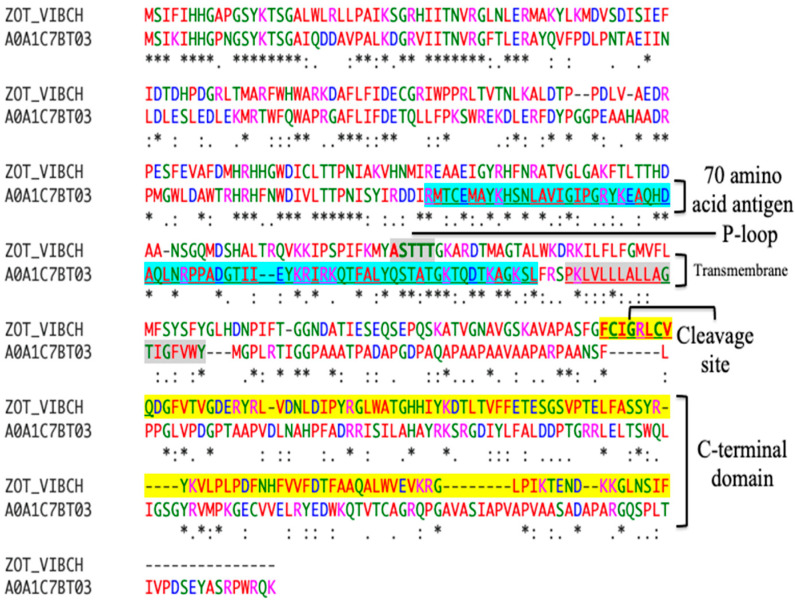
Annotative comparison of Zot, between *V. cholerae* and *P. aeruginosa*, reveals that our newly identified antigen is associated with the transmembrane domain. Stretch of 70 amino acid antigen (blue shading) is associated with the outer transmembrane (gray shading) of *P. aeruginosa*. C-terminus contains the cleaved toxicogenic fragment (yellow shading). A P-loop domain (gray shading) is found in *V. cholerae* to be present within the newly identified “high value” antigen. [Zot_VIBC, *V. cholerae*; A0A1C7BT03, *P. aeruginosa*]. Amino acid color alignments are based according to similarities in amino acid properties. [green, polar uncharged; magenta, positively charged; red, hydrophobic; blue, negatively charged]. [conserved identical sequence (*); conserved mutation (:); semiconservative mutation (.)].

Published reports reveal that signal peptides and transmembrane domains are characterized by exceptionally high epitope densities [67,68]. Accordingly, our annotation studies comparing *V. cholerae* and the *P. aeruginosa* Zot protein sequence reveal that our newly identified stretch of 70 amino acids is associated with the outer transmembrane of both species (Figure 4). A protein sequence comparison reveals a high degree of homology in the N-terminal domain of Zot. Data show that *V. cholerae-* and *P. aeruginosa*-encoded Zot proteins share 39% sequence homology and 54% positive sequence identities. Additionally, our analysis reveals the presence of a P-loop domain located within our target antigen (Figure 4). The presence of a conserved walker A motif, within our “high value” antigen, strongly suggests a functional role in transmembrane ATP binding and hydrolysis. Interestingly, our results also show that the cleavage site of the *V. cholerae* C-terminal domain is not present in the *P. aeruginosa* Zot protein, strongly indicating an alternative proteolytic processing for *P aeruginosa*-encoded Zot due to niche-associated conditions (Figure 4). Taken together, our results reveal that our “high value” vaccine candidate is localized to the transmembrane and characterized to exhibit high immunogenic potential. In the following section, the high epitope density of our antigen will be assessed using bioinformatic techniques and corroborated by the high percentage of identified epitopes in the IEDB (immune epitope database).

The generation of a protective immune response is highly dependent on the immunogenicity of an antigen. Immunogenicity refers to the degree of an antigen eliciting an immune response, which ranges from short-term immunity to long-term protection against a pathogen. Published reports reveal that the full-length, recombinant *V. cholerae* Zot protein is highly immunogenic. The intranasal immunization of mice with native recombinant Zot induces a very robust protective immune response, with approximately a 40-fold increase in serum immunoglobulin G (IgG) titers. These data also show that recombinant Zot stimulates high IgA titers in serum, as well as vaginal and intestinal secretions [69,70].

**Figure 5:**
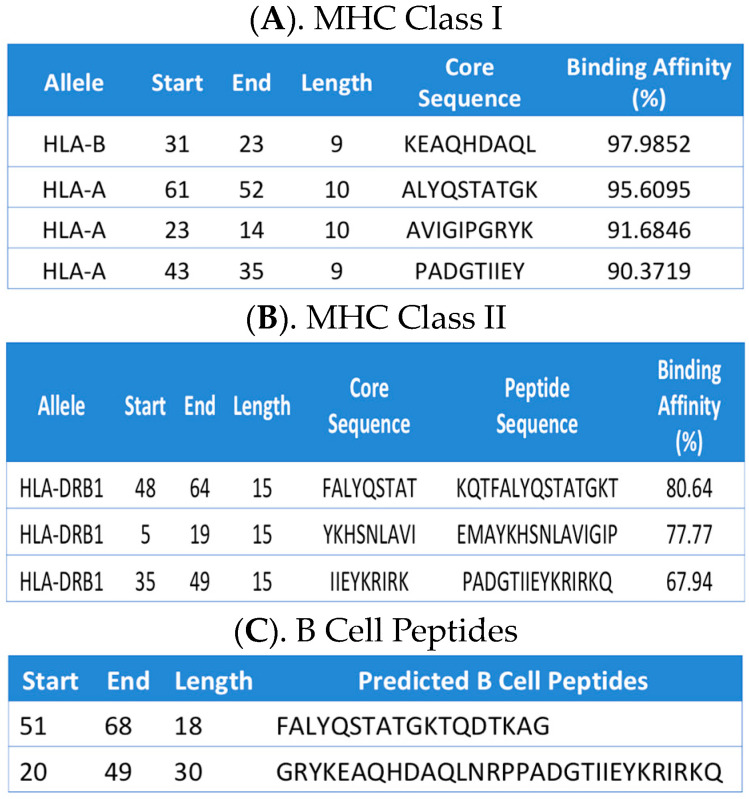
Using the Immune Epitope Database (IEDB), we have identified several high-affinity epitopes capable of targeting multiple *P. aeruginosa* genetic variants. These tables display the sequences extracted from the 70-amino acid “high value” antigen input sequence. Each row in this table corresponds to one peptide binding prediction. MHC-1 (**A**); MHC-II (**B**); B-cell Peptides (**C**). The columns contain the allele that the prediction was made for, the position of the peptide in the input sequences, the length of the peptide, the peptide sequence, and the predicted affinity.

To assess the immunogenic potential of our newly identified antigen, we performed a series of computational studies to determine the binding affinity of our intermembrane-associated stretch of 70 amino acids in the context of both MHC classes and a B cell receptor/antibody. By utilizing the Immune Epitope Database Analysis (IEDA) algorithm, we identified the presence of several, non-overlapping stretches of amino acids with very high immunogenic potential. In the context of MHC-I, we identified four distinct epitopes with very high binding affinity, ranging from 90% to 97% (Figure 5A). Additionally, our results show that these four epitopes do not overlap and are widely distributed throughout the 70-amino acid antigen. The analysis of MHC-II specific epitopes revealed the presence of three different non-overlapping regions of 15 amino acids with binding affinities between 68% and 80% (Figure 5B). Lastly, the analysis of our antigen shows two distinct regions with a high potential for serving as B cell receptor/antibody binding peptides. Advantageously, these two newly identified B cell peptides also span different regions of the same 70-amino acid antigen (Figure 5C). Taken together, our data further confirm the immunogenic potential of our newly identified “high value” antigen specific for multiple *P. aeruginosa* genetic variants.

**Figure 6:**
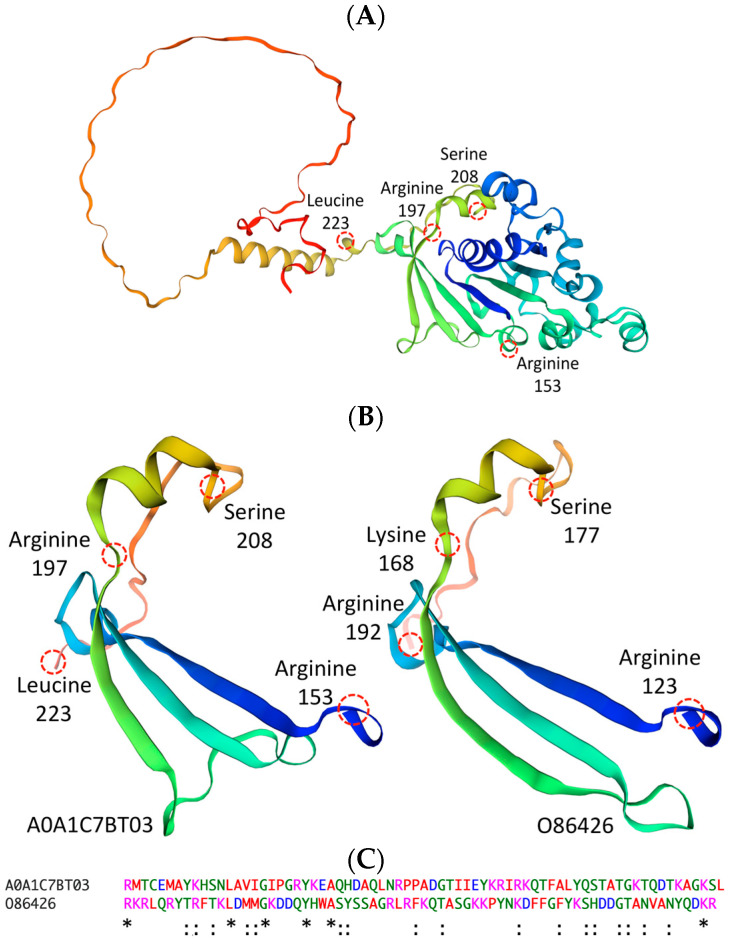
The newly identified evolutionary conserved stretch of 70 amino acids adopts a highly stable tertiary structure. Representation of native Zot protein encoded by genetic variant A0A1C7BT03 (**A**). Structural comparison of newly identified antigen between two of the least homologous *P. aeruginosa* strains (A0A1C7BT03 vs. O86426) show that they are analogous to each other by adopting the identical structural configuration (**B**). Sequence homology between Zot encoded A0A1C7BT03 and O86426 is estimated to be approximately 18% (**C**). Amino acid color alignments are based according to similarities in amino acid properties. [green, polar uncharged; magenta, positively charged; red, hydrophobic; blue, negatively charged]. [conserved identical sequence (*); conserved mutation (:); semiconservative mutation (.)].

An antigen’s conformational integrity is of crucial importance for high receptor/antibody binding affinity and proper intracellular trafficking from the initial uptake to antigen processing and cell surface display by antigen presenting cells (APC). The inherent structural characteristics have a great impact on antigen immunogenicity and the polarization of a robust immune response. Structural stability enhances the vaccine potency by increasing the rate of antigen proteolysis, antigen presentation and the induction of both humeral and cell-mediated responses. To assess the structural properties of our newly identified “high value” antigen, we used the Swiss-Model algorithm. First, we localized the conserved linear intermembrane-associated stretch of 70 amino acids to be situated between arginine 153 and leucine 223 of the native Zot protein (genetic variant: A0A1C7BT03). In the native form, our structural data show that amino acids between arginine 153 and arginine 197 adopt three antiparallel β strands, within a β-pleated sheet, and amino acids ranging from isoleucine 197 to serine 208 display a short helical configuration (Figure 6A). A comparative analysis of the complete native Zot protein versus our 70-amino acid antigen reveals a very high degree of structural homology. The newly identified intermembrane-associated antigen adopts the same antiparallel β-pleated sheet pattern and small helical conformation as can be observed in the native form. Furthermore, the structural comparison of the evolutionary conserved stretch of 70 amino acids among two of the least homologous strains (A0A1C7BT03 vs. O86426) show them both adopting the same identical conformation, at different amino acid positions relative to the native protein (Figure 6B). The quantitative assessment of the protein sequence homology between the two genetic variants reveals a percentage identity of 18% (Figure 6C). The adoption of identical conformations with such a high degree of sequence heterogeneity between the two strains continues to stress the importance of our newly identified 70-amino acid “high value” therapeutic antigen.

## 3. Discussion

Decade-long varying selective pressures have led to the vigorous global rise in a plethora of multidrug-resistant (MDR) Gram-negative bacteria (GNB). The lack of innovation directed at identifying new classes of antimicrobials is threatening the efficacy of the limited number of antibiotics currently used in clinical practice. International antimicrobial drug-development pipelines have become stagnant and have not kept on par with the inevitable reemergence of MDR pathogens [71]. This is beginning to undermine the worldwide healthcare systems, as more pathogens become extensively drug resistant while less novel antimicrobials are making it to market [72]. The intrinsic evolution of drug resistance against conventional antibiotics drives their limited lifespan and prompts the need to develop treatment modalities that mitigate the establishment of MDR [73]. At the current pace, soon, interventions such as basic surgical procedures and immunosuppressing chemotherapies would be accompanied by the increased risk of a life-threatening infection.

The genera *Pseudomonas* are a type of ubiquitous Gram-negative bacteria that commonly occupy soil and aquatic niches. Of the many different types of *Pseudomonas*, the species that is the leading cause of nosocomial GNB infections in humans is called *Pseudomonas aeruginosa*, a well-known hospital-acquired superbug that readily causes bacteremia, pneumonia, wound, burn and surgical site infections in both immunocompetent and immunocompromised individuals. *P. aeruginosa* infections are the leading cause of morbidity and mortality in patients afflicted with cystic fibrosis. *P. aeruginosa* harbors a very large genome (5.5–7 Mbp), and its relative size compared to other pathogens such as *Bacillus subtilis* (4.2 Mbp), *Escherichia coli* (4.6–5.3 Mbp) and *Mycobacterium tuberculosis* (4.4 Mbp) enables it to harbor significantly more virulent genes responsible for making it highly adaptable when interacting with a host [74,75,76,77]. To establish an aggressive infection, *P. aeruginosa* deploys different cell surface appendages to mediate physical host/pathogen interaction, enhances its quorum sensing capabilities, biofilm formation and the secretion of virulence factors [31,32,46,78,79]. Zot is one of several virulence factors secreted by *P. aeruginosa*. It is an evolutionary conserved toxin found in numerous GNB pathogens; these include, but are not limited to, *P. aeruginosa*, *N. gonorrhoeae*, *N. meningitidis*, *A. baumannii*, *K. pneumoniae*, *E. coli*, *S. enterica*, *Y. pestis* and *V. cholerae*. To date, Zot homologs have been identified strictly in GNB, without any trace in Gram-positive bacterial genomes. Zot’s association with the outer membrane of GNB is the restricting factor in its evolutionary path.

Zot is an outer membrane protein originally identified in the toxigenic Gram-negative bacteria, *Vibrio cholerae*. In the context of host–pathogen interaction, Zot serves as an enterotoxin capable of increasing intestinal epithelial tissue permeability [22,24,30]. Several reports show that the filamentous phage, CTXΦ, whose genome encodes a local stretch of several virulence factors (including the Zot gene), is responsible for mediating the horizontal transfer of this pathogenicity island to *V. cholerae* [23,25,26,27,80]. Interestingly, the high transmissible occurrence of the Zot gene along with ctxAB genes suggests their synergistic role in causing diarrhea typical of cholerae [24,80,81]. For example, the synergistic effect between Zot and cholerae toxin (CT) is displayed by the necessity of a detached epithelial cell rounding morphology (induced by Zot), for the maximal expulsion of electrolytes mediated by CT and the subsequent onset of acute dehydrating diarrhea commonly seen with *V. cholerae* infections [82].

Physically breaching epithelial cell barriers has profound effects on human health and disease, specifically in the context of disseminating infectious agents such as *P. aeruginosa*. Epithelial cell monolayers represent the foundational tissue barriers of the body [1,2,3,4,5,6,7,8,9]. Dermal abrasions, respiratory, enteric, oropharyngeal and genital mucosal epithelia are the primary portals of entry for most pathogens. Upon entry into a host, disease progression from a local infection to systemic dissemination requires the ability of the microbe to transmit across the mucosal epithelium and metastasize to distal anatomical sites. Studies show that the distribution of Zot receptor(s) coincides with the different permeating effects of the toxin on various types of epithelial tissue [22,24,29,83]. It is a bifunctional toxin responsible for enabling GNB pathogens to modulate their own paracellular transport between epithelial cell monolayers. Zot’s tight junctional regulatory properties are reversible, time and dose dependent, as well confined specifically to epithelial tissue [28]. Mechanistically, the binding of Zot to specific epithelial cell surface receptors initiates a signal transduction cascade that causes the disassembly of TJs, allowing the pathogen paracellular access to the basolateral host tissue [22,23,26,84]. In addition to its tight junctional regulatory properties, it has also been reported that Zot can regulate the post-translational modifications of cytoskeletal proteins [17,26,28]. The toxin induces the modifications of cytoskeletal complexes strategically localized to regulate the paracellular pathway between epithelial cells [26,82]. For example, ventilator-associated *P. aeruginosa* infections first establish a local pneumonic infection, where it promotes the accelerated decline in pulmonary function, followed by the subsequent paracellular passage between alveolar epithelial tissue (most likely mediated by Zot) and transition to a bacteremia state, where it disseminates throughout the lymphatic and/or circulatory systems causing severe sepsis [85,86,87,88,89].

It has become increasingly difficult to treat *P. aeruginosa* with conventional antibiotics. Its remarkable capacity to resist numerous antibiotics and use very high-level virulence mechanisms to counter antimicrobials and host defenses has made it imperative that alternative treatment strategies be developed to combat fatal *P. aeruginosa* infections [31,90,91]. In the context of infectious diseases, immunotherapy is a well-suited treatment modality that mitigates resistance, by harnessing a patient’s own immune defense mechanisms to fight disease more effectively [92,93,94]. It is well established that antibodies play a central role in the humeral immunity induced by vaccination, supported by T_H_ cells [95,96]. Antibody responses are generally essential in preventing the establishment of disease [97,98], whereas, in recent years, it has become evident that cytotoxic T cells also play a major role in fighting an infection, by controlling and clearing an already established disease [99,100]. For example, there is ample evidence showing that individuals with inherited or acquired antibody deficiencies have an increased susceptibility to the acquisition of infection, whereas T-cell deficiency results in the failure to control a pathogen after an infection [101,102,103]. Treatment options such as native and passive immunization have made it possible to prevent or treat both infection cycles from occurring [93]. In either case, a “high value” antigen is required to induce a multilevel immune response that includes the terminal differentiation of various cells of the lymphoid origin responsible for driving both humoral and cell-mediated immune responses.

“High value” antigens are immunogens capable of polarizing both branches of the lymphoid linage, followed by the establishment of a long-lasting immune memory, with the minimal number of administrations. The key features of “high value” therapeutic antigens are that they are long enough to carry multiple, non-overlapping, high-affinity epitopes dispersed throughout the antigen and are rigid enough to enhance binding affinities to B-cell receptors and secreted antibodies. Compared to the structurally undefined antigens, a “high value” antigen has a considerable length to adopt a stable configuration. Furthermore, it is essential that the therapeutic coverage of the antigen span the maximum number of genetically diverse strains. Using proteomic-driven comparative phylogenetics, in conjunction with structural modeling, we have traced the horizontal passage of the virulence factor, Zot, and its relatedness among various GNB. Through our work, we report the identification of a “high value” intermembrane-associated stretch of 70 amino acids capable of inducing both B- and T-cell responses with high affinity towards a multitude of different *P. aeruginosa* genetic variants. The characterization of this antigen has revealed it to possess four non-overlapping MHC I epitopes with binding affinities above 90%. In the context of MHC II epitopes, our results reveal the identification of three non-overlapping antigenic determinants dispersed throughout the length of the antigen as well, with binding affinities above 70%. Lastly, the assessment of structural-based immunogenicity indicates that our newly identified transmembrane-associated 70-amino acid antigen adopts a stable β-pleated sheet conformation linked to a short helical configuration, further increasing its structural-based affinity to B-cell receptors and antibodies.

In silico techniques are becoming a crucial part of the drug discovery process. These bioinformatic tools provide us with the potential for predicting novel therapeutic targets, while minimizing the number of false positives during the drug development process. The clinical relevance of such an approach is that it enables us to employ phylogenetics to identify “high value” vaccine candidates for the treatment of various infectious diseases [104]. For example, due to the large number of different *P. aeruginosa* genetic variants, our in silico method identified a highly conserved region that is shared among all the different strains and not just a few targets. Our data suggest that reverse vaccinology can be used to identify cross-species antigens for therapeutic purposes [61,62,64]. This technique can not only be applied to bacteria, but also to other types of pathogens that share homologous ancestral origins [104,105,106,107].

The rapid emergence of pan-drug resistance amongst numerous bacterial pathogens has outpaced the development of novel antibiotics, and as a result, has led to our continued efforts to identify cutting-edge, antibiotic-independent treatment strategies. The in silico prediction of drug targets has emerged as a promising technology platform for biomedical research and development [106,108]. The extensive homology of Zot, shared amongst numerous GNB pathogens, serves as an ideal target for the development of multi-strain-driven vaccines for *P. aeruginosa* and numerous other GNB pathogens. The advantage of employing comparative proteomics, in conjunction with structural modeling, is that it has allowed the identification of an evolutionary conserved intermembrane-associated stretch of 70 amino acids that is structurally identical among all the *P. aeruginosa* genetic variants queried, irrespective of how homologous the sequence is between the different strains.

Our reverse vaccinology strategy, using a combination of proteomic-driven bioinformatic tools in conjunction with structural modeling, has the potential to be used as a universal vaccine design technique that spans many different pathogens such as bacterial, fungal, viral and parasitic. The combination of phylogenetics and structural drug design methods will pave the way for the identification of “high value” antigenic drug leads that can be used to protect against numerous different species of pathogens and their genetic variants [104,105,106,107,108]. Future perspectives include the generation of monoclonal antibodies raised against our newly identified “high value” vaccine candidates and assess their binding affinities in vitro and therapeutic efficacy in vivo. 

## 4. Methods

### 4.1. UniProt Database Mining

The UniProt Knowledgebase provides users a high-quality and comprehensive set of protein sequences annotated with functional information. To fully understand the distribution of the Zot gene among GNB pathogens, we performed an extensive UniProt search with Zonula occludens toxin as the query (accessed 26 February 2024). Of the 284 predicted individual protein entities identified, the closest full-length matches (≥345 amino acids) to the *V. cholerae* Zot protein sequence were chosen. A total of 150 different protein entities and 35 species-specific bacteriophages were identified and chosen for our studies (Table 1). All the identified protein entities were distributed among eight different clinically relevant GNB pathogens: *P. aeruginosa*, *V. cholerae*, *N. gonorrhoeae*, *N. meningitidis*, *A. baumannii*, *K. pneumoniae*, *E. coli*, *S. enterica* and *Y. pestis*. It should be noted that the database mining results did not reveal the presence of Zot-like proteins associated with any Gram-positive bacteria [109,110].

### 4.2. NCBI BLAST and MUSCLE Algorithm

First, a tree was calculated by employing the MUSCLE algorithm, which counts the number of short sub-sequences (known as k-mers, k-tuples or words) that two sequences have in common, without constructing an alignment. In the second step, MUSCLE uses the tree to construct a progressive alignment. At each node of the binary tree, a pair-wise alignment is constructed, progressing leaves towards the root. The first alignment is made from two sequences. Later alignments included one of three different types: sequence–sequence, profile–sequence or profile–profile, where “profile” means the multiple alignment of the sequences under a given internal node of the tree. In conjunction with MUSCLE, we employed the Multiple Sequence Alignment Viewer Application (MSA), which is a web-based application that displays the graphical alignments generated by algorithms such as CLUSTALW, MUSCLE and alignments from NCBI BLAST. We viewed the alignments using the FASTA files generated by MUSCLE, UniProt and NCBI [65,111,112].

### 4.3. Backtranslation

In order to phylogenetically compare the nucleotide sequence homology among all the tested species-specific Zot protein sequences, a backtranslation approach was used. Backtranslation is a method of decoding an amino acid sequence into its corresponding codons. However, the degeneracy of the genome makes backtranslation very ambiguous, since multiple codons can represent the same amino acid. The most common strategy to overcome such hurdle is based on the imitation of codon usage within the target species. Accordingly, all the Zot protein sequences were backtranslated according to the species-specific codon optimization tools such as EMBOSS Backtranseq [113,114].

### 4.4. Immune Epitope Database and Analysis Resource (IEDB)

The IEDB was created to assist biomedical researchers in the development of new vaccines, diagnostics and therapeutics. The Analysis Resource is freely available to all researchers and provides access to a variety of epitope analysis and prediction tools. The tools include validated and benchmarked methods to predict MHC class I and class II binding. The predictions from these tools can be combined with tools predicting antigen processing, TCR recognition and B-cell epitope prediction. In addition, the resource contains a variety of secondary analysis tools that allow the researcher to calculate epitope conservation, population coverage and other relevant analytic variables. Predictions were obtained by using the 70-amino acid consensus sequence from strain A0A1C7BT03 [115,116].

### 4.5. SWISS-MODEL

SWISS-MODEL is an algorithm used for the automated comparative modeling of protein structures. In the “first approach mode” only an amino acid sequence of a protein is submitted to build a 3D model. Template selection, alignment and model building are completely automated by the server. In the “alignment mode”, the modeling process is based on a user-defined target–template alignment. The user specifies which sequence in the given alignment is the target sequence and which one corresponds to a structurally known protein chain from the ExPDB template library. The server will build the model based on the given alignment. Complex modeling tasks can be handled via the “project mode” using DeepView (Swiss-PdbViewer), an integrated sequence-to-structure workbench [117,118].

## Figures and Tables

**Table 1:** A total of 126 protein entities and 38 species-specific bacteriophages were identified in UniProt and distributed among 8 different gram-negative bacterial (GNB) pathogens. Different UniProt accession numbers distributed according to species.

*S. enterica*	*P. aeruginosa*	*A. baumannii*	*N. meningitidis*	*V. cholerae*	Phage
A0A379QXT6	A0A0H2Z826	A0A009IFH5	A0A0A8FBH3	ZOT	A0A0D5YII2
A0A3J8T5J6	A0A1B1XUY5	A0A009IPH3	A0A0G4BXF2	A0A059TZ50	A0A6H2UVW1
A0A3W0DS33	A0A1C7BC45	A0A0D5YHH3	A0A0G4BXV5	A0A0K1W029	A0A6H3SDW9
A0A3Y5WWD0	A0A1C7BT03	A0A0J0ZVM8	A0A0T7L458	A0A0N9E639	A0A7L5SKQ7
A0A5I0BDG5	A0A1C7BWJ9	A0A1S2G480	A0A0U1RHW9	A0A1X9T534	A0A7Z7KXF7
A0A5I3CR81	A0A1I1WSE1	A0A241YA97	A0A0U1RJQ1	A0A2I7YRQ5	A0A8B4N0A0
A0A5T2VLE5	A0A241XLX8	A0A2G1TM67	A0A112F978	A0A4D6FVS8	G1P_BPF1
A0A5U8J4F5	A0A367M4I3	A0A432AJV1	A0A425AZJ7	A0A655SGS8	G1P_BPFD
A0A5W2LVK4	A0A3S0IVW1	A0A5K1MT91	A0A828RN06		G1P_BPI22
A0A5W2LW37	A0A509J6G3	A0A5N5XT91	A0A828RNW2	*E. coli*	A0A1X3J1Y8
A0A5W8MDN4	A0A509JD43	A0A5P9QRB6	A0A9N7GF23		A0A4U9TGC6
A0A5X4GHS6	A0A5E5R224	A0A6F8TJW6	A1KR73	A0A2I5SNC4	Q1RBR0
A0A5X8YFW4	A0A643IWK8	A0A6H2UVW1	A9M0A6	A0A3Y3VCH3	A0A1D3HER3
A0A5Y0WW12	A0A6B1YIK7	A0A6H3EC82	E0N6U2	A0A3Y4Y4J3	A0A378VUX0
A0A5Y2U2W8	A0A6B1YIL4	A0A6H3ECH2	E3D282	A0A444R958	A0A6L2V467
A0A5Y2VPS7	A0A6M3UYA0	A0A6I4HQF2	E3D468	A0A8S7FTM0	B4RJA3
A0A5Y6EM93	A0A7L5EYC0	A0A7U3Y721	E6MVZ6	A7ZLV6	A0A0H5DLY7
A0A5Z4EMG4	A0A7M2ZLS6	A0A7U4DGM0	E6N078		A0A0H5E090
A0A608IEZ3	A0A7M2ZMC8	A0A809JHA0	Q9JRY6	*N. gonorrhoeae*	A0A0Y6T6A1
A0A618GAJ0	A0A844NQT0	A0A829K5W9	Q9JY47		A0A112FLQ9
A0A619A930	A0A894X9T7	A0A829K9J2	X5ENZ1	D6H5P6	A0A2X1UNH5
A0A619ACH0	A0A9Q9JRM6	A0A854NAT1		D6H5Q0	VG301
A0A619ADY4	B3G270	A0A858S3M3		Q5F6B3	VG424
A0A636GBL7	B3G280	A0A858S5Q5		Q5F6B6	A0A6B9J7K7
A0A6X6T3T2	B3G2P0	A0A8B5UBY1		Q5F7K6	A6VCB1
A0A715R1U3	C6JW42	A0A8I0FA89			Q56VN9
A0A720CYE7	O86426	A0A9Q2E630		*K. pneumoniae*	A0A0F6SIJ2
A0A729IWD7	Q58CI5	A0A9Q8M6J8			A0A0N9H972
A0A734CIM4	Q9I5K2	B0V4D4		A0A486UF20	A0A650EUI2
A0A741P1A0		B0V4E4		A0A8H9ZV07	A0A7D0JI66
A0A743NZG4		D0CAJ5		A0A9Q8EXA1	A0A8E4CGG1
A0A743PB40					A0A9E8MB84
A0A743PEF2				*Y. pestis*	F1CC69
A0A744EPC7					G8IRV1
A0A750EF42				A0A9P2VZ72	Q64EV0
A0A750HM75					Q8W6D9
A0A750MS12				Human	Q0WEP2
A0A751YXV3					
A0A757Y0U0				Q16625	
A0A759HB29				Q07157	
A0A759NSW2				Q9UDY2	
A0A760RQA5				O95049	
A0A974QHR0					

## Data Availability

The original data presented in the study are openly available in Uniprot database. All accession numbers are posted in materials section.
